# Sexual health literacy and preventive behaviors among middle-school students in a rural area during the COVID-19 situation: A mixed methods study

**DOI:** 10.34172/hpp.2022.22

**Published:** 2022-08-20

**Authors:** Mereerat Manwong, Saowanee Thongnopakun, Yuvadee Rodjarkpai, Aimutcha Wattanaburanon, Sawitree Visanuyothin

**Affiliations:** ^1^College of Medicine and Public Health, Ubon Ratchathani University, Ubon Ratchathani, Thailand; ^2^Faculty of Public Health, Burapha University, Chon Buri, Thailand; ^3^National Health Security Office (NHSO), Nakhon Ratchasima, Thailand

**Keywords:** Sex education, Reproductive health, Teenage pregnancy, Adolescent, SARS coronavirus 2

## Abstract

**Background:** This study explored the association between sexual health literacy (SHL) and preventive behaviors of pregnancies and sexually transmitted diseases (STDs) among middle-school students during the COVID-19 outbreak to aid in the development of an informative program.

**Methods:** Mixed-method study with stratified two-stage cluster sampling was used to obtain 730 students from 20 middle schools in a rural province of Thailand. Online self-administered questionnaire was used to collect data from February 2020 to February 2021. Two brainstorming sessions were conducted with a purposeful sample of 25 stakeholders. Multiple linear regressions were used to assess the relationships.

**Results:** The associated significant factors with the preventive behaviors were sex (b=2.06, 95% CI: 1.07, 3.05), nightlife (b=-2.33, 95% CI: -3.99, -0.67), drinking alcoholic beverages (b=2.24, 95% CI: -3.24, -1.23), sexual intercourse experience (b=-2.64, 95% CI: -4.56, -0.72), and SHL (b=0.12, 95% CI: 0.08, 0.16). The stakeholders recommended an edutainment online program via Facebook.

**Conclusion:** This study investigated factors associated with SHL and preventive behaviors among middle school students. The most effective variable in this model was SHL. Therefore, a trial of an online program emphasizing SHL improvement should be examined for effectiveness among these students and seems appropriate, considering their rural area context and middle-school status.

## Introduction

 Human immunodeficiency virus (HIV), viral hepatitis, and sexually transmitted diseases (STDs) are prevalent, and at least one million cases are reported annually worldwide. More than 2.3 million people die yearly from these causes.^1^ Worldwide, adolescents in particular also face sexual and reproductive health problems, such as STD risks, insufficient sexual health education and information, and unintended pregnancy.^2^ The annual estimated number of females aged 15–19 years who become pregnant is 21 million in developing countries and 12 million in low to middle income countries.^3^ In the United States of America, two-fifths of high school students have had sexual intercourse experience, one-fifth have had alcohol/drug use prior to sexual intercourse and use condoms, according to 2019 data. Youths aged 15–24 years account for half of all new STDs.^4^ In Thailand, although the pregnancy rate of adolescents aged 10–14 years was ≤10 per 1000, the rate for adolescents aged 15–19 years in 2020 was 28.7 per 1000. The STDs rate among those aged 15–24 was from 89.5 to 172.3 per 100 000 from 2011 to 2020.^5^ A surveillance report made by the reproductive department in 2021 showed a 77% unintended pregnancy rate.^6^ In one province covered by this study, the highest rate of 107.8 per 100 000 was reported for pregnancy and STDs and a high rate of 37.1 for teenage pregnancy.^6,7^

 Sexual health is crucial for physical and mental health and well-being, and it is fundamental in the prevention of unwanted pregnancy and STDs among adolescents worldwide.^1,2,8,9^ During the developmental changes of adolescents, sexual risk behaviors increase, particularly unprotected sexual intercourse, which results in STDs, HIV, teenage pregnancy, and other negative health conditions.^10,11^ Many strategies, techniques, and tools have been provided to prevent teenage pregnancies and STDs. Condoms provide the most effective way to prevent these problems.^12^ While low health literacy may lead to teenage pregnancy and STDs,^13^ sexual health literacy** (**SHL) is an essential way to decrease these problems.^14^ The COVID-19 pandemic has greatly affected students worldwide as a result of school closures. Many students have spent more time on media and online platforms, resulting in sexual risk behaviors that may lead to poor sexual reproductive health consequences.^15,16^ Thai student addiction to smartphones has been reported to be 35.9%.^17^ Therefore, Thai students as a group are particularly vulnerable to sexual risk behaviors, especially in the COVID-19 era. Furthermore, the family institution has also been shaken by this situation, which has caused socioeconomic problems, aggravating students’ exposure to the virtual world. Consequently, this circumstance brought more sexual risk behaviors to students than previously.^18^ Even though there has been a previous study about sexual risk behaviors in a province in Thailand, there have been limited studies about SHL among students, especially in rural areas, during the COVID-19 situation.Furthermore, there have been few methods for preventing teenage pregnancies and STDs among students during this pandemic. Therefore, this study was conducted to explore therelationship between SHL and preventive behaviors of pregnancy and STDs among middle school students. In addition, a program was developed based on the SHL survey results and ideas summarized from brainstorming. The results of this study offer an appropriate program to prevent teenage pregnancy and STDs among students during the COVID-19 era.

## Materials and methods

###  Study design, population sampling, and tools

 This was a mixed methods study with two phases conducted from February 2020 to February 2021 in a rural province of the Eastern region, Thailand. The study aimed to determine the relationship between SHL and the prevention of pregnancies and STDs among middle school students. The selected province is an important agricultural producer of fruits in Thailand’s economy.^19^

###  Phase 1 quantitative phase 

 In the first phase, there were a total of 4653 Grade 8 students from 71 schools during the 2019 academic year.^20^ An appropriate sample size had been calculated according to Wayne et al^21^ with α = 0.01, *p* = 0.51,^22^ and d = 0.05. The estimated required sample size (n) was 730 middle school students, after allowing 10% for missing data. A stratified two-stage cluster sampling method proportional to size was employed to recruit the sample. First, two public schools were randomly selected from the 10 districts in this province. Second, intact classrooms were randomly selected until an adequate sample size was obtained. Students were randomly recruited if they met the inclusion criteria: (1) Thai nationality; (2) stayed and studied in the area ≥3 months; (3) obtained permission from their parents, classroom teacher, and principal; and (4) currently in Grade 8. An exclusion criterion was pregnancy for a female student.

 The self-administered questionnaire consisted of six parts with 87 items as follows: (1) sociodemographic characteristics (10 questions, such as sex, cumulative grade point average [GPAX], daily income, etc); (2) sexual behaviors (15 questions, such as have boyfriend/girlfriend, nightlife, alcoholic drinking, sexual intercourse, etc); (3) communication about sex with parents (7 modified Likert scale questions)^23^; (4) influence of friends and media (10 modified Likert scale questions)^24,25^; (5) SHL (30 modified Likert scale questions, including accessing, understanding, appraising, and applying)^26,27^; (6) preventive behaviors of unintended pregnancy and STDs (15 modified Likert scale questions ).^27^ The self-administered questionnaire was managed via a universal resource locator (URL) and took 30 minutes. The item objective congruence (IOC) of the questionnaire, which was 0.60–1.00, was verified by five experts.^28^ The reliability of this questionnaire was tested with 30 students who had similar characteristics to the sample population; the overall Cronbach’s alpha coefficient statistic was 0.82.^29^

###  Phase 2 qualitative phase

 In the second phase, this study used purposive sampling for 25 stakeholders; 5 school health teachers who responded for promote and prevent student health in school, 10 grade 8 female students (who was not pregnant) and 10 grade 8 male students who lived in the district with the highest rate of teenage pregnancy. All stakeholders were invited for two brainstorming sessions.

 Brainstorming is “a situation where a group of people meet to generate new ideas and solutions around a specific domain of interest by removing inhibitions. People are able to think more freely, and they suggest as many spontaneous new ideas as possible. All the ideas are noted without criticism, and after the brainstorming session, the ideas are evaluated.”^30^ There have been many studies that have utilized brainstorming as a tool for the creation of interventions.^31,32^

 In this study, semi-structured questionnaires were used in two brainstorming sessions. Four issues were identified for situation analysis in the first brainstorming session: (1) SHL meaning, (2) the situation of pregnancy and STDs, (3) cause of pregnancy and STDs, and (4) impact of pregnancy and STDs. The second brainstorming session was used for program design, which focused on four issues: (1) previous solutions for pregnancy and STDs, (2) recommended solutions for these problems, (3) your roles in pregnancy and STDs prevention, and (4) the roles of other stakeholders in preventing these problems. Additionally, the IOC of the brainstorming questionnaire was 0.80 for students and 0.80–1.00 for teachers, which were obtained from five experts.

###  Analysis approaches

####  Quantitative 

 Individual data were analyzed by descriptive statistics: in numbers and percentages for categorical data and median (interquartile range [IQR]), minimum, maximum, mean, and standard deviation (SD) for continuous data. The univariate analysis was used to investigate association of each variable with preventive behavior of pregnancy and STDs such as independent *t* test, one-way ANOVA, and Pearson’s correlation coefficient. According to the univariate analysis, if the variable was found to be significant (*P*< 0.20), these variables were entered into the multivariate analysis. All assumptions of multiple linear regression were tested. The importance assumptions revealed that there was no multicollinearity and autocorrelation as following; (1) multicollinearity: variance inflation ratio test was 1.01–1.08. (2) autocorrelation: Durbin–Watson test was 1.51. The *P* value less than 0.05 was considered as a significant, and the confidence interval was calculated at the 95% level. The stepwise technique was used to analyze the factors associated with preventive behavior of pregnancy and STDs. The data analysis used IBM SPSS Statistics version 23x86 of the Burapha University licensed.

####  Qualitative

 A descriptive content analysis procedure was applied to examine the qualitative data. The data were recorded in a transcript, validated into categories according to similarity, and analyzed by two coresearchers. The brainstorming data were recorded and summarized by the researcher and research assistants. Afterwards, a program was developed for students with information intended to prevent pregnancy and STDs.

## Results

 The response rate in the co-creational survey was 100% for the online questionnaires. The results of the survey “Sexual health literacy among middle-school students in a rural area during coronavirus disease 2019 situation in Thailand” were divided into two topics: (1) factors associated with pregnancy and STDs prevention behaviors and (2) a summary of results from brainstorming and proposed an appropriate online program to prevent teenage pregnancy and STDs among students in the COVID-19 era. Both are described further in this section.

###  Factors associated with pregnancy and STDs prevention behaviors

 Two-thirds of the respondents were female students. The grade point average median was 3.0 (IQR = 0.9). The median of their daily income was 60 baht (IQR = 50); almost all of the students had adequate income. Two-thirds had married parents. Most of their parents worked in agriculture and were self-employed. More than 85% reported having very good or good relationships with their parents (Table 1). Most of the students were heterosexual, and one-third had a boy or girlfriend. More than half of them drank alcohol. Approximately 7.3% of the students reported having sexual intercourse experience. Additionally, the average score of comfortably communicating with their parents about sex issues, influence of friends and media, and SHL were 20.30 (SD = 5.16), 35.91 (SD = 4.93), and 96.81 (SD = 12.80), respectively. However, preventive behaviors of pregnancy and STDs had a mean of 50.93 (SD = 7.10) (Table 2). The univariable analysis found that sex, occupation of parents, have boyfriend/girlfriend, sexual intercourse experience, alcoholic drinking, nightlife, comfortable communication about sex with parents, and SHL were statistically significantly associated with preventive behaviors of pregnancy and STDs (*P*< 0.05) (Table 3). The results of the multiple linear regression revealed that SHL, sexual intercourse experience, nightlife, alcoholic drinking, and sex were significantly associated with preventive behaviors of pregnancy and STDs (*P* < 0.05). The values in the preventative behaviors model are shown in Table 4, which was used to predict the outcome variable of 11.4%. SHL was the most directeffective variable in the preventative behaviors of pregnancy and STDs in the model. If adjusted by sexual intercourse experience, nightlife, alcoholic drinking, and sex factors, it was found that a score of SHL increased the effect of preventive behaviors of pregnancy and STDs by 0.119 (Table 4).

**Table 1:** Number and percentage of demographic characteristics (n = 730)

**Demographic characteristics**	**Number**	**Percent**
Sex		
Male	298	40.8
Female	432	59.2
Cumulative grade point average (GPAX), Median (IQR)	3.0 (0.9)	
Daily income (baht), Median (IQR)	60 (50) Min = 10, Max = 200	
Daily adequate income		
Adequate income and saving	293	40.1
Adequate income	396	54.2
Inadequate income	41	5.7
Parents’ marital status		
Married	439	60.1
Separated	91	12.5
Divorced	182	24.9
Widowed	18	2.5
Occupational of parents		
Agricultural	290	39.7
Civil servant	39	5.3
Self-employed	313	42.9
Merchant	88	12.1
Relationship between student and parents		
Very good	293	40.1
Good	333	45.6
Moderate	90	12.3
Low	14	1.9

**Table 2:** Factors associated with pregnancy and STDs prevention behaviors (n = 730)

**Factor**	**Number**	**Percent**
Have boy/girlfriend		
Have boy/girlfriend but not staying together	237	32.5
Have boy/girlfriend but staying together	10	1.4
Have not had boy/girlfriend	483	66.1
Nightlife		
Ever	75	10.3
Never	655	89.7
Alcoholic drinking		
Ever	403	55.2
Never	327	44.8
Sexual intercourse experience		
Ever	53	7.3
Never	677	92.7
Communication about sex with parents		
(Mean, SD)	20.30 (5.16)	
Influence of friend and media		
(Mean, SD)	35.91 (4.93)	
Sexual health literacy (Mean, SD)	96.81 (12.80)	
Level of sexual health literacy		
High (≥102 score)	232	23.7
Moderate (90 - 101 score)	325	44.5
Low ( < 90 score)	173	31.8
Preventive behaviors of pregnancy and STDs (Mean, SD)	50.93 (7.10)	
Level of behavior to prevent pregnancy and STDs		
High (≥40 score)	239	32.7
Moderate (20–39 score)	265	36.3
Low ( < 20 score)	226	31.0

**Table 3:** Univariable analysis of factors associated with preventive behaviors of pregnancy and STDs

**Factor**	***P*** ** value**
Sex	< 0.001^a^
Parents’ marital status	0.529^b^
Occupational of parents	0.039^b^
Relationship between student and parents	0.914^b^
Have boy/girlfriend	0.003^a^
Sexual intercourse experience	0.002^a^
Alcoholic drinking	< 0.001^a^
Nightlife	< 0.001^a^
Comfortable communication about sex with parents	0.001^c^
Influence of friend and media	0.145^c^
Sexual Health Literacy	< 0.001^c^

^a^ Independent t test, ^b^ One-way ANOVA, ^c^ Pearson’s correlation coefficient.

**Table 4:** Multiple linear regression analysis toward preventing pregnancy and STDs

**Factors**	**b**	**SE**	**β**	**t**	**95% CI of b**	***P *** **value**
Sex (female)	2.059	0.505	0.143	4.076	1.07, 3.05	< 0.001*
Nightlife	-2.330	0.845	-0.100	-2.756	-3.99, -0.67	0.006*
Alcoholic drinking	-2.236	0.513	-0.157	-4.360	-3.24, -1.23	< 0.001*
Sexual intercourse experience	-2.638	0.978	-0.097	-2.697	-4.56, -0.72	0.007*
Sexual health literacy	0.119	0.020	0.215	6.075	0.08, 0.16	< 0.001*

Constant = 39.833; R^2^ = 0.120; R^2^_adj _= 0.114; F = 19.809; *P* value < 0.001. * Statistically significant.

###  Summary of results from brainstorming and proposed appropriate online program to prevent pregnancy and STDs among students in the COVID-19 era

 Almost all the students and teachers had not heard about SHL. Even though there was a class for sex education in school, students were urged to focus more on physical changes, safe abortions, and how students and families dealt with teenage pregnancy. The students thought that the sexual education class was boring and would be better if the teachers incorporated games, rewards, and entertainment methods into classes. The main source of sexual consultation was friends and the Internet; they also expressed a preference of using Facebook rather than Line. They recommended creating a program to prevent teenage pregnancy and STDs and via an online forum during the COVID-19 situation.

 The teachers also thought that sexual education was very important for their students, and online teaching methods with clear communication offered more benefits than the usual classroom for their students. The teachers suggested setting up consultation channels for students by health personnel. However, they believed that their students had a low level of sexual risk behaviors. For instance, they thought condoms were considered a toy by their students whenever they came to receive condoms during sexual health teaching in the classroom (Table 5).

**Table 5:** The summarization of the seven main activities offered for teachers and students

**Activity name**	**Online activity components**
Clear by Doctor	Brainstorm/group discussion by using infographic Searching game for reliable sources and rewards
Sex Need to Know	Watch sexual physical change animation Draw picture of individual change and send back to compete and receive rewards
Condoms Matter	Watch condom animation Buddy game to create quotes for using condoms and to receive rewards
Help!!! I’m Not Ready	Watch VDO about teenage pregnancy and consequences Brainstorm to solve the problem
Believable	Watch doctor VDO about myths and facts of sexual issues Question and answer via application and receive rewards
My Value	Watch “Say No” animation Discuss the pros and cons of “Say No” techniques Rehearsals of using different scenarios
My Choice	Work in group to create phrases or quotes for providing inspiration to each other’s aim to prevent pregnancy and STDs

## Discussion

###  Sexual reproductive health and associated factors between SHL and preventive behaviors of pregnancy and STDs

####  Sexual reproductive health during the pandemic 

 The COVID-19 pandemic offered the United States an opportunity to reexamine sexual reproductive health delivery to hard-to-reach and vulnerable populations, such as youths. Providers can increase accessible sexual reproductive health services by removing traditional barriers to care that youths routinely encounter. Encouraging self-initiated products, such as condoms, self-administered hormonal contraceptive injections, online platforms that mail oral contraceptive pills, and home-based sexual transmitted infection (STI) screening and treatment kits, could empower youths to engage in their health care to prevent unintended pregnancies and untreated STIs.^33^ Rapid advances in telehealth have increased virtual (phone and video) healthcare services and allowed many youths access to confidential and private virtual care. Immediate access to contraception is particularly important during the current pandemic, when the implications of COVID-19 on maternal and fetal well-being are not clearly understood. Video platforms enable providers to take detailed medical and sexual histories, assess STI risk, make a diagnosis based on the identification of a group of symptoms (i.e., syndromic management), prescribe contraceptives, and presumptively treat the most serious organisms responsible for producing a group of symptoms. The syndromic management of STIs in adolescent females is not without risks.^34^ A close follow-up to assess the resolution of symptoms is required.^33^ Virtual visits can also be used to triage patients for in-person visits as required for services, such as the placement of a long-acting reversible contraception (e.g., an implant or intrauterine device) or evaluation of lower abdominal pain. Although telehealth offers significant opportunities to serve youths, experiences during this pandemic also provide critical insight into its limitations as we begin to understand the characteristics of youths for whom telehealth is not feasible or acceptable. Physical and resource constraints may limit the use of telehealth among youths, and there may be conditions and symptoms for which virtual visits may not be appropriate or sufficient. These insights can guide how sexual and reproductive health care services for youths may adapt and evolve over the course of this pandemic because access to in-person clinical services may be reduced over the months or years to come.^33^

###  Associated factors between SHL and preventive behaviors of pregnancy and STDs among middle school students 

 The results revealed that three-fourths of the students had low to moderate levels of SHL. Two-third of the students had low to moderate levels of behaviors to prevent pregnancy and STDs. The significant factors associated with behavior to prevent pregnancy and STDs were sex, nightlife, alcoholic drinking, sexual intercourse experience, and the SHL score.

 Two-thirds of the students had low to moderate levels of behaviors to prevent pregnancy and STDs. Most of the students in this study could not avoid privately staying with their boyfriend or girlfriend and watching pornography together. Only 9.7% of the students reported avoiding watching pornography together with a boyfriend or girlfriend. Pornography was an important source for adolescents in studying sexual health; however, sexual intercourse, including for the first time, was related to watching pornography. Unplanned sexual intercourse is an expression of sexual risk behavior.^35,36^

 Female students had higher scores for preventive behaviors of pregnancy and STDs than male students (b = 2.059). Thai females have been encouraged to be very reserved. They have been suppressed by society not to openly express their sexuality because they are too young to be responsible for their sexuality and bodies.^37,38^ Although the Thai social structure lets males lead females, a female student will be stigmatized by society if she becomes pregnant.^38^ She and her family would have to bear the additional economic problems. She would also bear the burden of quitting school, pregnancy complications, etc.^39,40^

 Students who experienced nightlife had lower scores for preventive behaviors of pregnancy and STDs than did students who did not experience nightlife (b = -2.330). A study revealed that adolescents who preferred electronic dance music nightclubs were more likely to not use condoms during sexual intercourse and were more dependent on substances.^41^ Going into entertainment spaces provides students with easy access to drinking alcohol and taking other stimulating substances. They can lose self-control and safe sex inhibitions.^36,42^ Nightlife leads students to be at risk for unwanted pregnancies and STDs.

 Students who drank alcoholic beverages had lower scores for preventive behaviors of pregnancy and STDs than did students who did not (b = -2.236). There have been previous reports that a huge number of adolescents have consumed alcohol, and more than half of them have experienced sexual intercourse under the influence of alcohol.^43^ Thus, alcoholic consumption is an important risk factor for sexual behavior. This study’s result is consistent with previous studies, which found that alcohol was an influencing factor in adolescent pregnancy.^40,44,45^ Alcoholic drinking is related to a heightened perception of sexual effects resulting in unsafe sex.^46^

 Students who had experienced sexual intercourse had lower scores for preventive behaviors of pregnancy and STDs than students who did not (b = -2.638 scores).More than half of the students who had sexual intercourse did not always use condoms, which is consistent with previous research.^47^ Moreover, the COVID-19 pandemic has obstructed condom accessibility.^48^ Therefore, this pandemic has caused a decline in condom use,^49^ resulting in an increase in pregnancies and STDs.

 There have been meta-analysis studies that presented the relationship between health literacy and health behavior among adolescents.^50^ In addition, a study of five countries reported sexual and reproductive health literacy related to teenage pregnancy.^51^ This study was consistent with the evolving concept of health literacy,^52^ as it found that students had an increase of 0.119 in the scores for preventive behaviors of pregnancy and STDs for every one-point increase in SHL. This means that the students who had higher scores for SHL were more likely to perform preventive sexual behaviors than those who had lower scores for SHL. It was also found that 76.3% of students had a low to moderate level of SHL. According to this study, 65.5% of students had inadequate sexual and reproductive health literacy.^53^ In contrast, a previous Thai study revealed that 55.1% of university students had high SHL.^54^

###  Programs designed to prevent teenage pregnancy and STDs in the COVID-19 era

 The online program was created based on the survey results and the outcome of the stakeholders’ brainstorming. Due to the significant association between preventive behaviors of pregnancy and STDs and SHL and sexual risk behaviors (scores of SHL, sexual intercourse experience, nightlife, alcoholic drinking, and sex) and the stakeholders’ lack of knowledge about SHL, the SHL concept was integrated into the summary of ideas from the brainstorming session to design an appropriate program. There were four domains of SHL (access, understand, appraise, and apply),^26,55^ and the students’ SHL levels were low to moderate in all of them. To improve the access domain, “Clear by Doctor” was the activity designated to increase the accessibility of sexual health information and services. To improve the understanding domain, there were three sessions: “Sex Need to Know,” “Condoms Matter,” and “Help!!! I Am Not Ready.” These provided a sufficient understanding of sexual health and services in practice for students. To improve the appraise domain, “Believable” comprised criticism of sexual health myths. To improve the application domain, there were two sessions: “My Value” and “My Choice.” The activities were decision-making practices for sexual health risk scenarios. Students mentioned that formal sex education was boring and that they would like to find sexual health learning more enjoyable. They also proposed online learning during the COVID-19 situation. Therefore, many methods (brainstorming, group discussion, watching video clips, games, incentives, drawing pictures, questions and answers, and rehearsals) and tools (infographics, videos, animations, and scenarios) to increase the SHL of students via Facebook were used in this program.

## Conclusion

 This study investigated factors associated with SHL and preventive behaviors among middle school students in a rural area during the COVID-19 situation in Thailand. An online program was developed based on the survey results and brainstorming. The results showed a low score for both SHL and behaviors to prevent pregnancy and STDs. The significant factors associated with preventive behaviors of pregnancy and STDs were sex, nightlife venue, drinking alcoholic beverages, sexual intercourse experience, and SHL. The most effective factor was SHL, which was the main concept integrated into the online program. The students and teachers suggested the use of an edutainment online program via Facebook with various methods and tools for this learning.

## Acknowledgments

 The researcher would like to express gratitude to all the informants, including the related staff of the Faculty of Public Health, Burapha University, for all their support.

## Authors’ contributions

 MM: Conceptualization, data curation, formal analysis, methodology, validation, visualization, writing original draft preparation, writing review & editing. ST: Conceptualization, data curation, formal analysis, investigation, methodology, project administration, resources, software, writing original draft preparation, writing review & editing. YR and AW: Supervision, writing original draft preparation, writing review & editing. SV: Conceptualization, methodology, supervision, writing original draft preparation, writing review & editing

## Funding

 No grants were involved in supporting this work.

## Ethical approval

 Based on the Helsinki Declaration, the ethics approval for this research was obtained from Burapha University (certification code: Sci 109/2562). The researcher and research assistants explained the research details to the participants, including receiving informed consent from teachers and the students with their parents’ permission. Their privacy was protected, and their data will be kept confidential.

## Competing interests

 The authors report no conflicts of interest in relation to this work.

## Disclaimer

 The authors claim that no part of this paper is copied from other sources
